# State- and Territory-Level Nursing Home and Home Health Care COVID-19 Policies and Disease Burden

**DOI:** 10.1001/jamanetworkopen.2024.7683

**Published:** 2024-04-22

**Authors:** Patricia W. Stone, Suning Zhao, Ashley M. Chastain, Uduwanage G. Perera, Jingjing Shang, Laurent Glance, Andrew W. Dick

**Affiliations:** 1 Center for Health Policy, Columbia University School of Nursing, New York, New York; 2 Department of Anesthesiology and Perioperative Medicine, University of Rochester School of Medicine, Rochester, New York; 3 Department of Public Health Sciences, University of Rochester School of Medicine, Rochester, New York; 4 RAND Health, RAND Corporation, Boston, Massachusetts

## Abstract

This cross-sectional study creates a dataset and dashboard of US state- and territory-level COVID-19 policies specific to nursing homes and home health care agencies.

## Introduction

The COVID-19 pandemic disproportionately affected older persons,^1^ many of whom were served by home health care agencies (HHAs) and nursing homes (NHs). The extent to which state- and territory-level COVID-19 policies reinforced or expanded federal policies is unknown. Building on the work of others,^2^ we created a dataset and dashboard of state- and territory-specific NH and HHA policies linked with community and NH COVID-19 burden for researchers and public health officials to evaluate policy efficacy.

## Methods

In this cross-sectional study, we used the Council of State Government’s 2020-2021 State Executive Orders website^3^ and comprehensive searches of state and territory government websites to identify state- and territory-specific policies enacted from March 1, 2020, to July 1, 2022. We collected start and end dates and categorized policies as general or specific to NHs, HHAs, or both. Policies were grouped into 5 categories with 38 subcategories as (1) preventing virus transmission (n = 18), (2) expanding NH and/or HHA capacity (n = 5), (3) relaxing administrative requirements (n = 5), (4) reporting COVID-19 data (n = 3), and (5) admission and discharge policies (n = 7) (eMethods in Supplement 1).

We linked these policy data with community-level^4^ and NH-specific COVID-19 burden (case and mortality counts)^5^ and entered data into Tableau Desktop, version 2023.2.^6^ We used a color gradient and circle size to visualize policy counts and COVID-19 burden, respectively. The interactive dashboard displays temporality with zoom capability of setting, policy, and COVID-19 burden.

This study was approved by the Columbia University Institutional Review Board, who waived the need for informed consent because the study was not deemed human participant research. We followed the STROBE reporting guideline.

## Results

**Figure 1.**
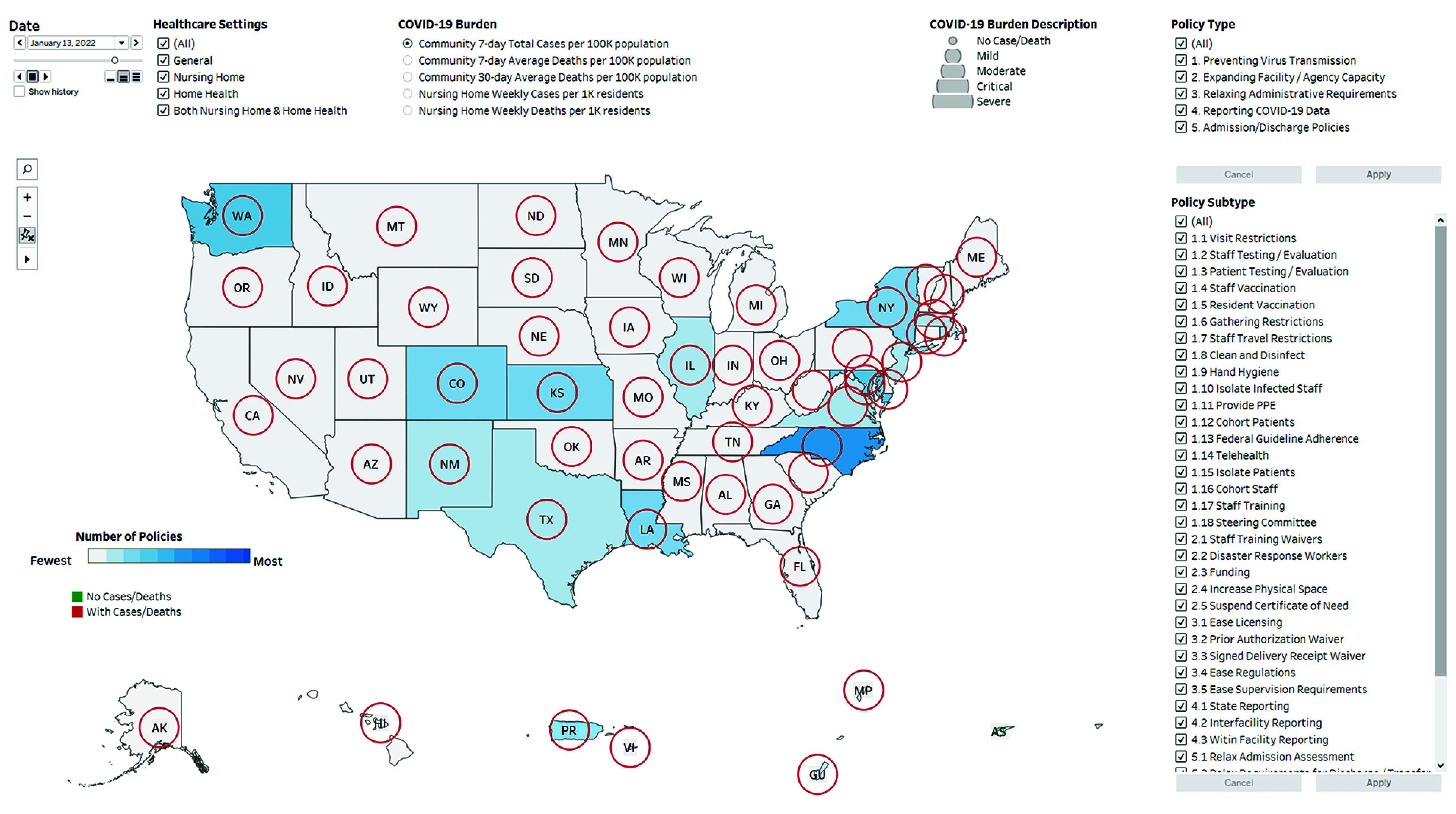
Overview of a National Dashboard of State- and Territory-Level COVID-19 Policies Aimed at Post–Acute Care Settings The date filter allows for daily selection of dates from March 1, 2020, to July 1, 2022. A slider is included under the date selection box for viewing policy progression throughout the pandemic. The health care settings filter consists of 4 checkboxes, allowing for the selection of target health care settings (general health care settings, nursing homes, home health care agencies, and both). Comprehensive definitions are found in the eTable in Supplement 1. The COVID-19 burden parameter consists of 5 checkboxes, allowing for the selection of 5 distinct categories of COVID-19 burden (cases and deaths) at the community and nursing home levels. The policy type filter allows for the selection of 5 broad policy categories. The policy subtype filter contains 38 distinct subcategories related to the broader categories. On the map, the number of policies is indicated by a color gradient, ranging from the least (light gray) to the most (dark blue). COVID-19 burden is depicted as circles of varying size, with larger diameters signifying increasing severity. Circles are red if there were deaths recorded during that period, green if there were no deaths, and gray if no data were available. The central US map can be enlarged for ease of viewing, while the 5 US territories remain fixed in size.

**Figure 2.**
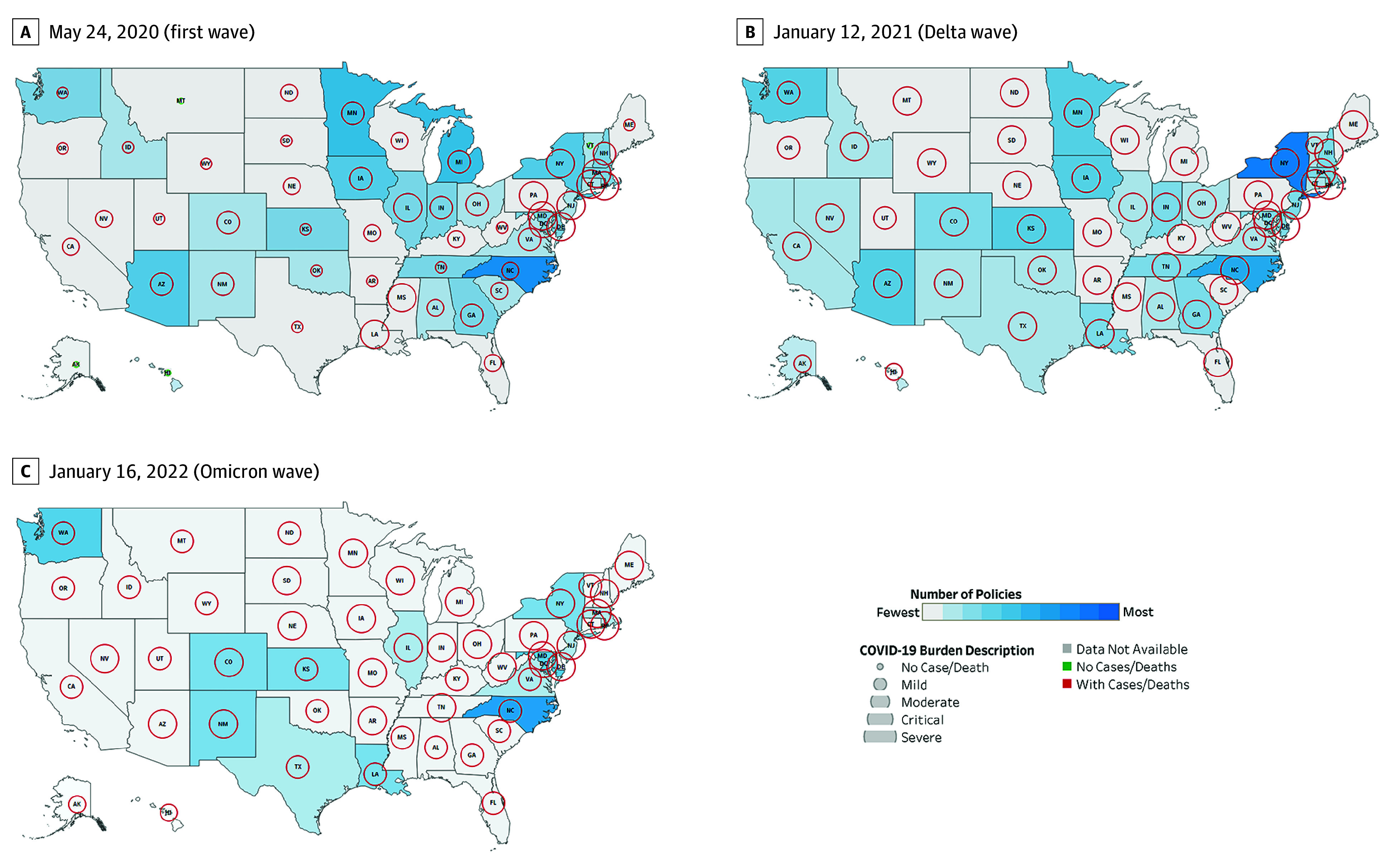
Number of Policies and COVID-19 Burden in the 50 US States and Washington, DC, at 3 Points During the Pandemic Maps depict May 24, 2020 (during first wave and after mandatory case and death reporting in nursing homes begins), January 12, 2021 (Alpha and Delta variants), and January 16, 2022 (Omicron variant). On the maps, the number of COVID-19 policies is indicated by a color gradient, ranging from the least (light gray color) to the most (darker blue color). All targeted health care settings (general, nursing homes, home health agencies, and both) were selected for this visual. COVID-19 burden is depicted as circles of varying size, with larger circles signifying increasing severity. The 7-day average of COVID-19 community deaths per 100 000 population was selected for this visual. Circles are red if there were deaths recorded during that period and green if there were no deaths.

We identified 1400 policies across 50 states and 5 territories. Most included all health care settings (n = 846), followed by NH-specific (n = 486), NH- and HHA-specific (n = 43), and HHA-specific (n = 25) policies. The most common policy category was preventing virus transmission (n = 736), followed by expanding NH and HHA capacity (n = 325), relaxing administrative requirements (n = 184), reporting COVID-19 data (n = 79), and admission and discharge (n = 54). The dashboard (Figure 1) illustrates variation in the number of policies per state and severity of COVID-19 burden indicated by color gradient and circle diameter, respectively. Figure 2 highlights the dynamic change in NH and HHA policies and COVID-19 burden. For example, on May 24, 2020, Montana, Hawaii, and Alaska had no COVID-19 deaths or policies, in contrast with North Carolina’s moderate burden and several policies. By January 12, 2021, New York had a severe COVID-19 burden and the greatest number of policies, while Pennsylvania, Montana, and Florida had a similar COVID-19 burden but fewer policies.

## Discussion

The dataset and dashboard described in this study are potentially important tools for researchers and public health officials and could provide a template for visual platforms that may inform future efforts to manage public health crises. Variations in COVID-19 burden and state and territory policy responses displayed in the dashboard highlight the complexity of pandemic management. Exploratory analyses demonstrated that higher numbers of policies at the state and territory levels were not consistently associated with reductions in community- or NH-level COVID-19 burden, suggesting policy effectiveness may depend on implementation and compliance. We also found limited attention to HHAs compared with NHs, despite both settings serving vulnerable older populations. This suggests a gap in public health planning, raising questions about resource allocation and prioritization among health care settings during pandemics.

Study limitations include the primarily descriptive data, underlying data from various sources, and limited evaluation of efficacy of public health policies on population COVID-19 outcomes. Future public health planning and pandemic responses should include adaptive and targeted policy interventions and should consider specific needs of all health care settings. Dashboards have the potential to help formulate data-driven decision-making during public health crises.

## Notes

Supplement 1. **eMethods.** Policy Dataset and Dashboard Development **eTable.** Definitions of Post–Acute Care COVID-19 Policy Categories and Subcategories
eReferences

Supplement 2.
Data Sharing Statement
