# Supplementary material for: State- and Territory-Level Nursing Home and Home Health Care COVID-19 Policies and Disease Burden
Source: JAMA Netw Open. 2024 Apr 22;7(4):e247683. doi: 10.1001/jamanetworkopen.2024.7683 (PMC11036140; doi:10.1001/jamanetworkopen.2024.7683)
Supplement: Supplement 1. — eMethods. Policy Dataset and Dashboard Development eTable. Definitions of Post–Acute Care COVID-19 Policy Categories and Subcategories eReferences [file jamanetwopen-e247683-s001.pdf]

Supplementary Online Content

Stone PW, Zhao S, Chastain AM, et al. State- and territory-level nursing home and home health care COVID-19 policies and disease burden. *JAMA Netw Open*. 2024;7(4):e247683. doi:10.1001/jamanetworkopen.2024.7683

eMethods. Policy Dataset and Dashboard Development

eTable. Definitions of Post–Acute Care COVID-19 Policy Categories and Subcategories

eReferences

This supplementary material has been provided by the authors to give readers additional information about their work.

© 2024 Stone PW et al. *JAMA Network Open*.

eMethods. Policy Dataset and Dashboard Development

All study procedures were approved by a single Institutional Review Board (sIRB); the collaborating institutions agreed to rely on the sIRB at the lead institution.

Policy Dataset Development

Policy Identification

For this dataset, the Council of State Government's (CSG) website, "2020-2021 State Executive Orders – COVID-19 Resources for State Leaders,"[1] was the initial source for identifying state/territory-based COVID-19 policies since the state/territory executive orders were already consolidated and classified. Firstly, post-acute care-related policies were identified under the CSG classification of "Assisted Living/Nursing Home/Long-Term Care Facilities". Building on the work of others,[2] these policies were then organized in our dataset under one of five broad categories: 1) preventing virus transmission, 2) expanding facility/agency capacity, 3) relaxing administrative requirements, 4) reporting COVID-19 data, and 5) admission/discharge policies. These policies were then further divided into smaller more specific subcategories (n = 38) guided by the policy intention (**eTable 1**).

A subsequent Google search was conducted, revealing limitations of the CSG website. The CSG website did not identify COVID-19 policies aimed at healthcare settings in general, as well as extensions and line-by-line modifications of already-identified policies. A detailed internet search was conducted for each U.S. state and territory (50 U.S. states, District of Columbia, Puerto Rico, U.S. Virgin Islands, Guam, Northern Mariana Islands and American Samoa). Policies collected previously from the CSG website were verified for accuracy and end dates of policies were added, considering any extensions. New policies that were identified during the search, as well as modifications to previously-collected policies, were added to the dataset. We also identified policies aimed at general healthcare settings (not naming nursing homes or home healthcare agencies specifically) that fit our predetermined categories and subcategories. Searches were conducted in the order of a state/territory's FIPS code using terms such as "[state/territory name]" in combination with "COVID-19 policies", "executive orders", "public health orders" and "governor office news briefings". Through these searches, we learned that individual states/territories have their own methods of organizing and archiving COVID-19-specific policies; some states/territories had very organized archives by date and are searchable by topic, while others removed any policies relating to a previous gubernatorial administration if an election had occurred. If policies were not archived on the official government site, we attempted to locate the policies in place during a previous administration on other websites or on the internet archive (via Wayback Machine).

Policy Categorization

Executive Orders and Public Health Orders provided specific information regarding which policies were enacted and what time period they were effect. Government Office News Briefings were useful in identifying policies for states that did not archive their orders; the briefings also provided additional context to specific political actions and their intended outcomes. Collectively, this information was used to create a dataset of policies addressing COVID-19 mitigation in post-acute care settings that takes into consideration policies aimed at healthcare settings in general and includes the actual date in which a policy is no longer in effect.

For each policy, the following data was collected in Microsoft Excel 2019: start date, end date, policy type, policy sub-type, application to healthcare setting(s), source, website link, and comments. The start dates of each policy were stated in executive orders and public health orders or when a new policy was enacted to replace a prior policy. The end dates of specific policies were listed based on their original end date. Policy extensions were added as a different row and highlighted to differentiate them from the original policy. This process followed until a final expiration date occurred due to either non-renewal of the policy or replacement by a new policy. Modifications were treated as new policies (new Excel row) if the wording of the policy changed appreciably. Furthermore, if a policy was allowed to expire and was not included in an extension within 2 weeks (allowing for any backdating), any subsequent extension was added as a new row and was treated as a new policy to account for the time gap. In the final dataset, the extensions were collapsed into the original policy, date ranges for each extension were added in a comment, and the original policy received a new end date. The application of the policy was determined as either "General", "Nursing Homes (NH) Only", "Home Healthcare Agencies (HHA) Only" or "Both NH & HHA". Policies that address healthcare settings as essential businesses, but do not explicitly mention nursing homes or home healthcare agencies were listed as "General". Policies that specifically address nursing homes were listed as

© 2024 Stone PW et al. *JAMA Network Open*.

“NH Only” and policies that specifically address home healthcare agencies were listed as “HHA Only”. Policies that address both nursing homes and home healthcare agencies were categorized as “Both NH & HHA”. One team member categorized and another team member confirmed the categorization; if categorization differed, discussion with other team members occurred until consensus was reached. The source was listed as the title of the specific policy or news briefing being referenced; a link to the source was also listed. Comments included a brief description of the specific policy that supports its categorization under a specific policy type and subtype. Overall, 1,400 policies were included in the final policy dataset.

Dashboard Development

Data Manipulation and Integration

The final policy dataset was imported into R 4.3.1.[3] The number of unique policies that were effective for each day within a state/territory was counted by each type, subtype and healthcare setting. This process resulted in a dataset that exclusively presented the number of policies for each subtype, type and healthcare setting by state /territory and date, from March 1, 2020, to July 1, 2022. Additionally, a new variable capturing the maximum counts of policies for each subtype, type and healthcare setting among dates and states/territories was also generated for visualization purposes.

To visualize relationships between COVID-19 burden and policy formulation and impact, five COVID-19-related outcomes were selected, encompassing both community-level outcomes and nursing home-level outcomes. Community outcomes served as indicators for assessing the overall COVID-19 trend and any associations between COVID-19 burden and policy making. These outcomes included 7-day total cases, 7-day average deaths and 30-day average deaths, which were calculated based on the state/territory-level COVID-19 database from the New York Times.[4] Furthermore, those outcomes were weighted based on 2020 population census data at the state/territory level,[5] resulting in variables that reflect community COVID-19 burden per 100,000 population. Nursing home-specific outcomes, including weekly nursing home cases or deaths per 1,000 residents, were aggregated to the state level using COVID-19 nursing home data from the National Healthcare Safety Network (NHSN)[6]; these outcomes assume the number of cases and deaths remain constant over seven days within one week. The COVID-19 outcome data were merged with the state/territory-level policy dataset by state/territory and date.

Dashboard Development in Tableau

The merged dataset was imported to Tableau Desktop 2023.2 for dashboard development. A dual-axis map sheet was formulated, within which the number of policies is represented by color. To better visualize the policy counts, we utilized a gradient of 10 colors, measuring deciles of policy counts relative to the maximum policy counts. Consequently, the color “grey” denoted the fewest policies, while “dark blue” represented the highest count of policies. The dashboard with the date slider allows users to view the trends in policy counts from March 1, 2020, to July 1, 2022.

COVID-19 burden is depicted as circles on the map. A parameter was created in Tableau to allow dashboard users to select one of the five outcomes: 1) total community-level COVID-19 cases per 100,000 population over 7 days; 2) average community-level COVID-19 deaths per 100,000 population over 7 days; 1) average community-level COVID-19 deaths per 100,000 population over 30 days; 4) weekly nursing home cases per 1,000 residents; and 5) weekly nursing home deaths per 1,000 residents. The severity of the burden was stratified into five categories, with an exclusive category for no cases or deaths. The rest was divided into quartiles that were categorized as “Mild”, “Moderate”, “Critical”, and “Severe”. The severity can be visualized by the diameter of the circle, with the largest indicating severe burden and the smallest denoting “no cases or deaths”. States/territories with “no cases or deaths” have green circles, those “with cases or deaths” have red circles, and those with “data not available” for nursing home outcomes before May 17, 2020 have grey circles. Four filters can be applied to the map, including date, healthcare setting, and policy type and subtype (organized in a hierarchy).

To construct the dashboard, the original map sheet was duplicated into six different sheets, each illustrating different regions: U.S. mainland, Alaska, Hawaii, Puerto Rico and Virgin Islands, Guam and Northern Mariana Islands, and American Samoa. These sheets were combined into one dashboard with four filters, the COVID-19 outcome parameter, and a legend. Users have the flexibility to view the map by date, healthcare setting, COVID-19 burden, policy type and policy subtype. This finalized dashboard was uploaded and published using Tableau Public, accessible at https://www.nursing.columbia.edu/PAC_Dashboard.

© 2024 Stone PW et al. *JAMA Network Open*.

eTable. Definitions of Post–Acute Care COVID-19 Policy Categories and Subcategories

Categories	Subcategories	Definitions of Sub-Categories
1. Preventing virus transmission<br><br>*Definition: Policies that identify or prevent COVID-19 exposure*	1.1 Visit restrictions	Limits or restricts visitors to nursing homes, with certain exceptions
	1.2 Staff testing/evaluation	Requires testing of nursing home staff for COVID-19
	1.3 Patient testing/evaluation	Requires testing of nursing home residents for COVID-19
	1.4 Staff vaccination	Requires vaccination of nursing home staff
	1.5 Resident vaccination	Requires vaccination of nursing home residents
	1.6 Gathering restrictions	Limit or restricts gathering within nursing homes
	1.7 Staff travel restrictions	Limits or restricts staff from travel in-state, out of state, and/or internationally
	1.8 Clean and disinfect	Requires the cleaning and disinfecting of nursing home facilities
	1.9 Hand hygiene	Recommends hand hygiene policies or reinforces current hygiene policies within nursing home facilities
	1.10 Isolate infected staff	Requires any staff with COVID-19 symptoms to stay home
	1.11 Provide PPE	Recommends the usage of masks, the preservation of PPE, and the acquisition of PPE
	1.12 Cohort patients	Requires residents that have tested positive or have unknown status to be separated from residents that have tested negative
	1.13 Federal guideline adherence	Reinforces adherence to public health guidelines on local and federal levels
	1.14 Telehealth	Allows or removes restrictions regarding the use of telemedicine in nursing homes
	1.15 Isolate patients	Requires quarantining of patients upon admission to nursing homes
	1.16 Cohort staff	Assigns a cohort of staff to care for residents that tested positive or have unknown COVID-19 status
	1.17 Staff training	Completion of additional training for nursing home staff to improve the pandemic response
	1.18 Steering committee	Requiring the development of advisory councils, comprised of nursing departments and certification boards, to prepare for infectious disease emergencies
2. Expanding facility/ agency capacity<br><br>*Definition: Policies that increase the workforce, available space, or funding*	2.1 Staff training waivers	Waives training and certification requirements in order to expand the workforce to address worker shortages in nursing homes
	2.2 Disaster response workers	Initiates the National Guard to address COVID-19
	2.3 Funding	Allocates funding to nursing home facilities
	2.4 Increase physical space	Expands the physical capabilities of a nursing home by being able to take in more patients, acquiring more beds, etc.
	2.5 Suspend certificate of need	Ability to temporarily establish home healthcare services at any location

© 2024 Stone PW et al. *JAMA Network Open*.

3. Relaxing administrative requirements<br><br>*Definition: Policies that ease the NH's ability to see patients*	3.1 Ease licensing	Eases requirements to maintain a license within the already existing workforce
	3.2 Prior authorization waiver	Waives prior authorization requirements, including the receipt of Medicaid payment
	3.3 Signed delivery receipt waiver	Waives requirements for signed receipts of medical equipment, devices, and supplies
	3.4 Ease regulations	Eases regulations in order to more easily provide care during COVID-19
	3.5 Ease supervision requirements	Supervisory visits of home health aides can be conducted remotely or indirectly
4. Reporting COVID-19 data<br><br>*Definition: Policies requiring the sharing of COVID related data*	4.1 State reporting	The reporting of COVID-19 data prior to the national mandate which only occurred in 4 states: Arizona, New York, Connecticut, Maryland
	4.2 Interfacility reporting	The reporting of COVID-19 data after the national mandate and between different facilities and hospital systems
	4.3 Within facility reporting	The reporting of COVID-19 data after the national mandate and within the same facility
5. Admission/Discharge Policies<br><br>*Definition: Policies addressing the transfer or discharge of patients*	5.1 Relax admission assessment	Relaxes requirements for a typical comprehensive assessment before admitting a patient to a nursing home facility
	5.2 Relax requirements for discharge/transfer	Eases typical requirements for discharging or transferring a patient from a nursing home facility
	5.3 Transfer protocol	The adopting of an infectious disease transfer protocol within the nursing home facility
	5.4 Prevent hospitalizations	The prevention of unnecessary hospitalizations to an acute care hospital to limit the drain on those hospital resources
	5.5 Suspend involuntary discharges	Prevents the involuntary discharge or transfer or patients from nursing homes to other facilities
	5.6 Cannot prohibit admission based on test results	Prevents the prohibition of admission or readmission of patients based on COVID-19 status
	5.7 Obtain negative test prior to admission	Hospitals are not allowed to discharge patients to a nursing home without obtaining a negative result

Note: PPE = personal protective equipment

© 2024 Stone PW et al. *JAMA Network Open*.

eReferences

1. The Council of State Governments. 2022-2021 Executive Orders: COVID-19 Resources for State Leaders. Accessed February 4, 2022. https://web.csg.org/covid19/executive-orders/.
2. Chen AT, Ryskina KL, Jung HY. Long-Term Care, Residential Facilities, and COVID-19: An Overview of Federal and State Policy Responses. J Am Med Dir Assoc. Sep 2020;21(9):1186-1190.
3. *R: A language and environment for statistical computing* [Software]. Version 4.3.1. Vienna, Austria: R Foundation for Statistical Computing, 2023.
4. The New York Times. Coronavirus (COVID-19) Data in the United States. Accessed October 16, 2023. https://github.com/nytimes/covid-19-data.
5. United States Census Bureau. 2020 Census. Accessed October 16, 2023. https://www.census.gov/programs-surveys/decennial-census/decade/2020/2020-census-main.html.
6. Center for Medicare & Medicaid Services. COVID-19 Nursing Home Data. Accessed October 19, 2023. https://data.cms.gov/covid-19/covid-19-nursing-home-data.

© 2024 Stone PW et al. *JAMA Network Open*.
