# Supplementary material for: State- and Territory-Level Nursing Home and Home Health Care COVID-19 Policies and Disease Burden
Source: JAMA Netw Open. 2024 Apr 22;7(4):e247683. doi: 10.1001/jamanetworkopen.2024.7683 (PMC11036140; doi:10.1001/jamanetworkopen.2024.7683)
Supplement: Supplement 2. — Data Sharing Statement [file jamanetwopen-e247683-s002.pdf]

Data Sharing Statement

Stone. State- and Territory-Level Nursing Home and Home Health Care COVID-19 Policies and Disease Burden. *JAMA Netw Open*. Published April 22, 2024.
doi:10.1001/jamanetworkopen.2024.7683

Data
Data available: Yes
Data types: Other (please specify)
Additional Information: Dashboard and technical documentation
How to access data: Dashboard and technical documentation will be publicly available at https://www.nursing.columbia.edu/PAC_Dashboard.
When available: With publication

Supporting Documents
Document types: Other (please specify)
Additional Information: Underlying data and data dictionary
How to access documents: Underlying data and data dictionary will be available by request at https://www.nursing.columbia.edu/PAC_Dashboard.
When available: With publication

Additional Information
Who can access the data: Researchers whose proposed use of the data has been approved. They will be able to fill out a form on https://www.nursing.columbia.edu/PAC_Dashboard to request underlying data and data dictionary.
Types of analyses: For any type of research.
Mechanisms of data availability: After a researcher fills out the online form and the information is reviewed by the research team, the requesting researcher will be sent the underlying data (via Excel) and data dictionary (Word).
Any additional restrictions: N/A.
